# Mini-Mental State Examination in patients with hepatic encephalopathy and liver cirrhosis: a prospective, quantified electroencephalography study

**DOI:** 10.1186/1471-230X-13-107

**Published:** 2013-07-02

**Authors:** Dorota Koziarska, Ewa Wunsch, Małgorzata Milkiewicz, Maciej Wójcicki, Przemysław Nowacki, Piotr Milkiewicz

**Affiliations:** 1 Department of Neurology, Pomeranian Medical University, ul. Unii Lubelskiej 1, 71-252, Szczecin, Poland; 2 Liver Research Laboratories, Pomeranian Medical University, Al. Powstancow Wlkp. 72, 70-111, Szczecin, Poland; 3 Medical Biology Laboratory, Pomeranian Medical University, Al. Powstancow Wlkp. 72, 70-111, Szczecin, Poland; 4 Department of General, Transplant and Liver Surgery of the Medical University of Warsaw, Warsaw, Poland

**Keywords:** Liver cirrhosis, Hepatic encephalopathy, Mini-mental state examination, Electroencephalography, Frequency analysis

## Abstract

**Background:**

Mini-Mental State Examination (MMSE) is one of the most commonly used methods in the assessment of cognitive mental status. MMSE has been used in hepatology but its usefulness in the evaluation of hepatic encephalopathy (HE) has never been properly assessed. The aim of the study was to investigate the value of MMSE in detection of HE in patients with cirrhosis.

**Methods:**

One hundred and one consecutive patients with liver cirrhosis underwent neurological examination, MMSE and electroencephalography (EEG). Spectral analysis of EEG was done with calculation of mean dominant frequency (MDF) and relative power of delta, theta, alpha and beta rhythms. Minimal HE was diagnosed in patients with normal neurological status and alterations in spectral EEG. Statistical analysis included Fisher’s exact and Anova analysis. Categorical data were compared using Levene’s test for equality of variances. Correlation-coefficient analysis was performed by the Pearson’s r or Z-test, as needed. Tests performance was assessed by the calculating the area under the ROC curve (AUC) and evaluating its difference from reference area (AUC=0.5). A *p* value <0.05 was considered statistically significant.

**Results:**

Overt HE was identified in 49 (48.5%) and minimal HE in 22 (21.8%) patients. Although there were significant correlations between both severity of liver disease (Child-Pugh classification), overt HE (West-Haven criteria) and various MMSE items, MDF showed no correlation with any of MMSE items as well as MMSE summary score. MMSE (score and items) did not discriminate patients without HE and minimal HE. The only significant differences between patients without HE and with overt HE were seen in respect of MMSE score (*p*<0.02), orientation to place (*p*<0.003), repetition (*p*<0.01) and complex commands-understanding (*p*<0.02). Test performance analysis has shown that MMSE has no value as a prediction method in determining minimal HE and in respect of overt HE has a sensitivity of 63% and specificity of 52% by a cut-off level at 27.5 points to diagnose overt HE.

**Conclusions:**

In conclusion, although MMSE score and single items are altered in patients with overt HE, MMSE has no value in the assessment of minimal HE. Because MMSE could be impaired in several cognitive dysfunctions, more specific test should be used for measuring HE.

## Background

Hepatic encephalopathy (HE) is a serious complication of liver cirrhosis negatively affecting both health-related quality of life and survival [1-3]. It comprises a plethora of neuropsychiatric symptoms ranging from subtle neurocognitive alterations to severe life-threatening neurological impairment [4]. Although it is now considered as a continuum of neurocognitive deterioration [5], HE is usually divided into minimal and overt HE, regarding the change in the mental state. A characteristic feature of minimal HE is lack of a detectable brain dysfunction in the clinical examination, however subclinical psychomotor slowing and cognition deficits are typically present in minimal HE and clinically relevant [6-8]. Overt HE is characterized by various symptoms encompassing neuromuscular abnormalities, intellectual functions, personality and consciousness, traditionally graded on four stages according to West-Haven criteria [3].

Several diagnostic methods were implemented both to diagnose and to monitor HE, but until now there is no consensus about the diagnostic standard. Mini-Mental State Examination (MMSE) has been also used in the assessment of the severity and in monitoring of HE in several studies [9-13]. MMSE is a brief questionnaire, that was introduced by Folstein*et al*. in 1975 for the assessment of cognitive mental status [14]. After minor subsequent modifications it is currently one of the most popular methods for the screening of cognitive function impairment used both in clinical practice and in research in broad variety of medical conditions [15]. MMSE evaluates neurocognitive deficits, that are also present in minimal HE and are forerunners of more severe HE. These are easily underestimated by a routine examination, therefore the information drew from the detailed analysis of MMSE could potentially serve as a well structured and objective way of identification of changes in mental status in patients with cirrhosis. Nevertheless the real usefullness of MMSE in the evaluation of HE has never been properly studied. Moreover MMSE was until now only interpreted as a total score, and was not divided into separate items. We therefore performed a detailed analysis of MMSE items and investigated the potential value of MMSE in evaluation of HE in patients with liver cirrhosis.

## Methods

### Patients

One hundred and one consecutive patients with liver cirrhosis: 68 (67.3%) males and 33 (32.7%) females, aged 54.5 ± 11.8 years (range 18–87) were enrolled to our study. Detailed demographic data are shown in Table 1. Included subjects were both in-patients and out-patients who received their treatment at our institution. Included patients had no history of factors impairing mental status, such as consumption of psychotropic drugs, active alcohol misuse, neurological (i.e. dementia, epilepsy, alcoholic seizures) and other than liver disease severe medical problems. Liver cirrhosis was diagnosed by liver biopsy and/or a typical appearance of the liver on abdominal ultrasound and/or computed tomography scan. Blood samples for liver biochemistry and venous ammonia level were collected on the day of examination. The Child–Pugh score was calculated to assess the severity of liver disease [16,17]. All patients underwent detailed neurological examination, MMSE and closed-eye electroencephalography (EEG) performed by one experienced neurologist (DK).

**Table 1:** Demographic data of study group

**Demographic data**	**Study group (n=101)**
**Age (years, mean ± SD, range)**	54.5 ± 11.8 (18–87)
**Gender (male/female)**	68 (67.3%)/33 (32.7%)
**Education years (mean ± SD, range)**	12.0 ± 2.9 (7–19)
**Etiology of cirrhosis**	
Alcoholic	38 (37.6%)
Autoimmune	21 (20.8%)
Viral hepatitis	19 (18.8%)
Other	20 (19.8%)
Mixed	3 (3.0%)
**Previous hepatic encephalopathy**	
Yes	34 (33.7%)
No	67 (66.3%)
**Child-Pugh class**	
A	44 (43.6%)
B	35 (34.6%)
C	22 (21.8%)
**Bilirubin (mg/dl, mean ± SD, range)**	3.2 ± 3.6 (0.3-21.2)
**INR (mean ± SD, range)**	1.3 ± 0.2 (1.0-1.8)
**Albumin (mean ± SD, range)**	3.6 ± 0.63 (2.2-4.9)
**Ammonia (mean ± SD, range)**	84.2 ± 98.5 (7.1-805)

### MMSE

MMSE consists of several short questions and problems grouped into 8 items: orientation to time and to place, registration, attention and calculation, recall, language skills, repetition and complex commands (i.e. write a sentence of his own choice, reading a sentence, understanding a command, copy of pentagons). Each item is scored and summary score of maximal 30 points is calculated with higher scores indicating better cognitive performance.

### EEG analysis

EEG was recorded for 20 minutes, with eyes closed, in a condition of relaxed wakefulness, using 18 silver-silver chloride electrodes placed according to the International 10–20 system (Nervus-Multimedia EEG, Taugagreininghf, Reykjavík, Iceland). The ground and the reference electrode was Fz. The impedance was kept below 5 kΩ. Each channel had its own analog-to-digit converter, the resolution was 0,19 uV/digit. Single observer (DK) visually inspected the traces and classified according to the Parsons- Smith grading [18] modified by Amodio *et al.*[19].

Spectral analysis was carried out on the transverse derivation P3-Pz and P4-Pz in the frequency range of 0,5-30 Hz. Pre-selected 80–100 s periods of artifact-free recording, split into 5 s epochs were analyzed by applying Fast Fourier Transformation. Mean dominant frequency (MDF) was calculated, the mean values were used. The relative power of delta (0.5-4.0 Hz), theta (4.0-8.0 Hz), alpha (8.0-13.0 Hz) and beta (13.0-30.0 Hz) rhythms were calculated. EEG alterations associated with HE were then graded using the thresholds proposed by Amodio *et al.*[20]. Grade 1 by MDF >6.8 and Theta% ≥35%; grade 2 by MDF ≤ 6.8 Hz and Delta% < 49%; grade 3 MDF ≤ 6.8 and Delta% ≥49.

### Diagnosis of HE

Clinical evaluation of overt HE was performed by an experienced neurologist (DK) and comprised of a detailed, comprehensive neurological examination according to West-Haven criteria. Following symptoms were particularly noted: psychomotor slowing, impaired performance of arithmetic, disorientation for time and place, inappropriate behavior, extrapyramidal signs, asterixis, ataxia etc. Patients were also reviewed in respect of neuropsychiatric disorders (e.g. epilepsia, alcoholic seizures) and circadian rhythm disturbance. Overt HE was diagnosed in patients, in whom typical abnormalities were present independent from EEG results. West-Haven criteria were applied for the assessment of the severity of overt HE [3,21]. Minimal HE was considered in patients with unaltered neurological status but with alterations in spectral analysis of EEG (i.e. grade ≥ 1 according to the system proposed by Amodio P. *et al.*) [20].

### Statistics

Patient measures are reported as means ± standard deviation (SD). All data were analyzed using Stat-View-5 Software (SAS Institute, Cary, NC, US) and included Fisher’s exact and Anova analysis. Categorical data were compared using Levene’s test for equality of variances. Correlation-coefficient analysis was performed by the Pearson’s r or Z-test, as needed. Tests performance was assessed by the calculating the area under the ROC curve (AUC) and evaluating its difference from reference area (AUC=0.5). A *p* value <0.05 was considered statistically significant.

### Ethics

Written informed consent was obtained from each patient included in the study. The study protocol was approved by the appropriate ethics committee of Pomeranian Medical University, Szczecin, Poland and conforms to the ethical guidelines of the 1975 Declaration of Helsinki (6th revision, 2008).

## Results

### MMSE results in the study group

All included patients, who completed MMSE performed three items: registration, language skills and complex command - reading a sentence fully correctly. These items were not further analyzed. MMSE (summary score and most of the items) were independent from age and gender of the patients. Only results for a complex command - copy of pentagons item were more altered in older patients (*p*<0.02, CI95% = −0.430 - -0.053). Moreover males had significantly better results in respect of a complex command - write a sentence than females (mean: 0.97 ± 0.37 *vs* 0.84 ± 0.175 points, *p*<0.03).

There were significant correlations between severity of liver disease (Child-Pugh classification) and MMSE summary score (r = −0.420, *p*<0.0001, CI 95% = −0.573 - -0.239) and several MMSE items: orientation to time (r = −0.476, *p*<0.0001, CI95% = −0.618 - -0.303), orientation to place (r = −0.391, *p*<0.0001, CI95% = −0.549 - -0.206) and complex commands: understanding a command (r = −0.295, *p*<0.005, CI95% = −0.468 - -0.099), write a sentence (r = −0.389, *p*<0.0001, CI95% = −0.547 - -0.203) and copy of pentagons (r = −0.353, *p*<0.0005, CI95% = −0.517 - -0.163). Patients in Child-Pugh class A and B had better results of MMSE (summary score and most of the items) than patients in Child-Pugh class C. In contrast patients in Child-Pugh class A and B achieved similar results in most of the MMSE items. These results are summarized in Table 2.

**Table 2:** MMSE results dependent of severity of liver disease (Child-Pugh classification)

**MMSE categories**	**Class A (n=44)**	**Class B (n=35)**	**Class C (n=22)**	**A *vs *B**	**A *vs *C**	**B *vs *C**
**MMSE summaryscore**	27.6 ± 1.9	27.0 ± 2.0	24.8 ± 4.8	*ns*	*p*<0.0005	*p*<0.01
**Orientation to time**	5.0 ± 0.2	4.7 ± 0.8	4.2 ± 0.9	*p<*0.05	*p<*0.0001	*p<*0.03
**Orientation to place**	4.9 ± 0.2	4.8 ± 0.4	4.4 ± 0.7	*ns*	*p*<0.0001	*p*<0.001
**Attention and calculation**	3.8 ± 1.4	3.7 ± 1.2	3.5 ± 1.9	*ns*	*ns*	*ns*
**Recall**	2.2 ± 0.9	2.4 ± 0.7	1.9 ± 0.8	*ns*	*ns*	*p<*0.05
**Repetition**	0.8 ± 0.4	0.8 ± 0.4	0.7 ± 0.5	*ns*	*ns*	*ns*
**Complex commands (Understanding)**	2.9 ± 0.3	2.8 ± 0.5	2.4 ± 1.0	*ns*	*p*<0.01	*p*<0.03
**Complex commands (Write a sentence)**	1.0 ± 0.0	0.9 ± 0.2	0.8 ± 0.4	*ns*	*p*<0.001	*p*<0.02
**Complex commands (Copy of pentagons)**	1.1 ± 0.6	0.9 ± 0.3	0.6 ± 0.5	*ns*	*p*<0.002	*ns*

*MMSE* Mini-Mental State Examination.

Venous ammonia concentration did not correlate with MMSE summary score and all items except the repetition item (r=−0.324, *p*<0.002, CI95%=−0.499 - -0.125).

### MMSE results in patients with HE

Neurological examination identified 49 (48.5%) patients with overt HE: 26 (25.7%) in grade 1, 16 (15.8%) in grade 2 and 7 (6.9%) in grade 3 according to West-Haven criteria. In 22 (21.8%) subjects without clinical symptoms of HE, minimal HE was diagnosed based on spectral analysis of EEG.

MMSE (score and items) did not discriminate patients without HE and minimal HE, however there were significant differences between patients without HE and with overt HE in respect of MMSE summary score (*p*<0.02), orientation to place (*p*<0.003), repetition (*p*<0.01) and complex commands - understanding (*p*<0.02) (Table 3). In patients with more severe overt HE according to West-Haven criteria we observed reduction in most of MMSE scores, which reached a statistical significance in the following items: orientation to place, attention and a complex command - write a sentence. MMSE summary score was reduced in patients with grade 3 of overt HE in comparison to patients with grade 1 HE (Table 4).

**Table 3:** MMSE results in patients without and with HE (minimal and overt)

**MMSE categories**	**Without HE (n=30)**	**Minimal HE (n=22)**	**Overt HE (n=49)**	**Without HE *vs *minimal HE**	**Without HE *vs *overt HE**	**Minimal HE *vs *overt HE**
**MMSE summaryscore**	27.7 ± 1.8	27.5 ± 2.0	25.9 ± 3.7	*ns*	*p*<0.02	*p*<0.05
**Orientation to time**	4.8 ± 0.8	4.9 ± 0.3	4.6 ± 0.9	*ns*	*ns*	*ns*
**Orientation to place**	4.7 ± 0.2	4.9 ± 0.2	4.6 ± 0.6	*ns*	*p*<0.003	*p*<0.01
**Attention and calculation**	3.7 ± 1.4	3.8 ± 1.5	3.6 ± 1.6	*ns*	*ns*	*ns*
**Recall**	2.4 ± 0.6	2.1 ± 0.9	2.1 ± 0.9	*ns*	*ns*	*ns*
**Repetition**	0.9 ± 0.3	0.8 ± 0.4	0.7 ± 0.5	*ns*	*p*<0.01	*ns*
**Complex commands (Understanding)**	2.9 ± 0.3	2.9 ± 0.2	2.6 ± 0.8	*ns*	*p*<0.02	*p*<0.02
**Complex commands (Write a sentence)**	1.0 ± 0.2	0.9 ± 0.2	0.9 ± 0.3	*ns*	*ns*	*ns*
**Complex commands (Copy of pentagons)**	1.0 ± 0.2	1.1 ± 0.9	0.8 ± 0.4	*ns*	*ns*	*p*<0.01

*MMSE* Mini-Mental State Examination, *HE* hepatic encephalopathy.

**Table 4:** MMSE results in patients with overt HE according to West-Haven criteria

**MMSE categories**	**Grade 1 (n=26)**	**Grade 2 (n=16)**	**Grade 3 (n=7)**	**Grade 1 *vs*2**	**Grade 1 *vs *3**	**Grade 2 *vs *3**
**MMSE summary score**	26.8 ± 2.7	25.4 ± 3.9	23.7 ± 5.6	*ns*	*p*<0.02	*ns*
**Orientation to time**	4.7 ± 0.6	4.4 ± 0.9	4.4 ± 1.1	*ns*	*ns*	*ns*
**Orientation to place**	4.8 ± 0.4	4.4 ± 0.8	4.4 ± 0.8	*p*<0.02	*p*<0.05	*ns*
**Attention and calculation**	4.0 ± 1.2	3.7 ± 1.6	2.1 ± 1.9	*ns*	*p<*0.003	*p<*0.02
**Recall**	2.2 ± 0.8	1.9 ± 0.9	1.9 ± 0.9	*ns*	*ns*	*ns*
**Repetition**	0.7 ± 0.5	0.6 ± 0.5	0.9 ± 0.4	*ns*	*ns*	*ns*
**Complex commands (Understanding)**	2.5 ± 0.7	2.6 ± 0.9	2.5 ± 1.1	*ns*	*ns*	*ns*
**Complex commands (Write a sentence)**	1.0 ± 0.2	0.8 ± 0.4	0.9 ± 0.4	*p<*0.05	*ns*	*ns*
**Complex commands (Copy of pentagons)**	0.8 ± 0.4	0.7 ± 0.5	0.6 ± 0.5	*ns*	*ns*	*ns*

*MMSE* Mini-Mental State Examination, *HE* hepatic encephalopathy.

Test performance analysis has shown that MMSE has no value as a prediction method in determining minimal HE (AUC = 0.43, *p* = 0.317, CI95%= 0.295 – 0.561). Although AUC analysis reached the statistical significance in respect of overt HE (AUC = 0.64, *p* = 0.018, CI95% = 0.528 – 0.752), and by a cut-off level at 27.5 points MMSE summary score had a sensitivity of 63% and specificity of 52% to diagnose overt HE.

### MDF

Mean MDF in study group was 7.2±2.3 Hz (range 2.3 - 13.2 Hz). MDF values were independent from subjects’ age, however were lower in males than females (mean MDF 6.7 ± 2.2 Hz *vs* 8.2 ± 2.4 Hz respectively, *p*<0.003) Although MDF correlated significantly with severity of liver disease (i.e. Child-Pugh classification, r = −0.373, *p*=0.0001, CI95% = −0.531 - -0.191) and HE (i.e. West-Haven classification, r = −0.337, *p*=0.0005, CI95% = −0.5 - -0.152), MDF values showed no correlation with any of MMSE items as good as MMSE summary score (data not shown).

## Discussion

The appropriate diagnosis of HE causes several problems. After exclusion of other causes of mental impairment, a careful clinical examination is usually sufficient for the diagnosis of overt, mainly more advanced stages of HE (grade 3 and grade 4 i.e. coma). However less advanced overt HE require a detailed neuropsychological evaluation. Moreover, for detection of minimal HE a clinical examination has a little value because of the lack of clear manifestations, and an additional more sensitive diagnostic tool is in this case necessary. However, until now no method is available that can discriminate the specific stages of HE objectively through the entire spectrum of minimal and overt HE [4]. The West-Haven grading system, which classifies HE into categories, remains criticized because of its intense subjectivity and low intra- and inter-observer reliability [5,22]. Therefore several other more objective diagnostic tools (e.g. neuroimaging, neurophysiological methods or psychomotor tests), were implemented to diagnose both minimal and overt HE, but there is still an ongoing debate which of them should be a diagnostic standard [23-25]. In several studies spectral analysis of EEG has been proven to be a valuable, sensitive and observer –independent method to detect minimal HE [19,26], therefore it can be used as a reference tool and we applied it in our study as well. However the EEG detects cortical neuronal activity and may not ideally reflect a wide variety of neurophysiological events seen in HE [24]. Moreover it is not broadly implemented in a daily practice because of its limited availability and the need of an experienced personnel to appropriate interpretation of the obtained electroencephalograms. Therefore there is still a need to develop screening tests for a clinical routine and for a research in studying patients with liver cirrhosis, which will cover a whole spectrum of pathophysiological spectrum of the phenomenon.

Neuropsychological diagnostic tools show a number of practical advantages making them potent candidates for the common use in HE assessment [27,28]. Generally they are simple to perform, not time-consuming can be used in the out-patient clinic. For this purpose both single paper-pencil tests (such as the block design test, the trail making test and the digit symbol test) as well as standardized batteries (i. a. the Repeatable Battery for the Assessment of Neuropsychological Status, the Wechsler Adult Intelligence Scale—Revised, the Auditory Verbal Learning, the Everyday Memory Questionnaire) have been implemented [29-35]. The complex tools have a superior value in comparison to single modalities because they cover a broad spectrum of neurocognitive deficits. However among plenty of these tests only the Psychometric Hepatic Encephalopathy Score (PHES) was especially constructed for detecting minimal HE [36]. Others have a great importance in the diagnosis of cognitive alteration in elderly or neuropsychiatric disorders, in the estimation of the effect of psychoactive drugs or to measure intelligence, but their value in the field of HE remains to be established.

MMSE is one of above mentioned complex neuropsychological tools, that was also occasionally used for identification patients with HE in clinical trials [9-13] and for several reasons could serve as a potential screening test for this purpose. MMSE assesses cognitive abilities: orientation (spatial and time), attention, concentration, calculation, immediate and short-term memory, visuo-spatial orientation, praxis and language skills [14]. Some of these functions are also altered in cirrhotic patients in the course of minimal HE and in stages 1 and 2 overt HE according to West-Haven criteria, long before neurocognitive decline is obvious and clinically easy to detect [4].

Indeed our study clearly shows that MMSE (summary scores and most of the items) is altered in patients with more advanced liver disease measured by Child-Pugh classification. Likewise we observed an increasing reduction of the most of MMSE items in patients with overt HE. However these differences reached the statistical significance only in few items and mostly in more advanced stages of overt HE (i.e. in grade 2 and 3) and the numeric difference between MMSE scores was small and unlikely to be clinically useful. These included orientation to place, attention and calculation and complex command - writing a sentence of own choice. Moreover the test performance analysis has shown that MMSE summary score has low both specificity and sensitivity in detection of overt HE. Furthermore MMSE summary score and all items did not differentiate patients with minimal HE from those without any signs of HE (i.e. with normal neurological state and without alteration seen in spectral EEG) and did not correlate with MDF values. These observations clearly shows that MMSE should not be considered an appropriate tool for detection of minimal HE and in overt HE in stage 1 and 2 according to West-Haven classification. Although in our study MMSE was altered in patients with stage 3 of overt HE, but this can be detect clinically and generally requires no additional method.

All included into our analysis subjects performed three items: registration, language skills and complex command - reading a sentence fully correctly. These observations can be explained by the heterogeneous nature of HE and by fact that cognitive functions are selectively compromised in this disorder [37,38]. While several cognitive functions are altered very early in the natural course of the disorder (i.e. attention, visuo-spatial orientation, psychomotor speed), some others are manifestations of more advanced HE (disorientation to place, lethargy, somnolence). On the other hand some intellectual functions such as language skills, long-term memory and intelligence are generally unaffected, even in patients at higher stages of HE [8,39].

## Conclusion

In conclusion, although HE is a disorder that affects the mental status and causes a decrease in the MMSE, our study strongly suggests that this modality is not an appropriate tool in defining an encephalopathy-related status of patients with cirrhosis and should not be used for this purpose in clinical trials. In view of the complex nature of HE there is still a need to develop more specific test for objective measuring through the entire spectrum of this phenomenon.

## Abbreviations

MMSE: Mini-Mental State Examination; HE: Hepatic encephalopathy; EEG: Electroencephalography; MDF: Mean dominant frequency.

## Competing interests

The authors declare that they have no competing interests.

## Authors’ contributions

DK designed the study, carried out the neurological examination, EEG analysis, performed MMSE and participated in the draft of the manuscript. EW participated in the design of the study, recruited participants and drafted the manuscript. DK and EW contributed equally in the work. MM performed statistical analysis. MW participated in the design and coordination of the study. PN conceived of the study, and participated in its design and coordination. PM participated in the design and coordination of the study and corrected the manuscript. All authors read and approved the final manuscript.

This study was presented at EASL Meeting, Barcelona, Spain 2012, and awarded with the Young Investigator Bursary.

## Pre-publication history

The pre-publication history for this paper can be accessed here:

http://www.biomedcentral.com/1471-230X/13/107/prepub
